# Working on the bias

**DOI:** 10.1007/s40037-017-0396-3

**Published:** 2018-01-02

**Authors:** Anna Harris

**Affiliations:** Faculty of Arts and Social Sciences, Maastricht University, Maastricht, The Netherlands

I used to make my own clothes, including neckties. With my friend and fellow medical student Mimi, we bought silk and learned how to make ties which we sold to other medical students needing to look more professional in the eyes of patients and the doctors teaching and assessing them. When making ties we learned to work on ‘the bias’. This means working with fabrics against the grain, or obliquely. This is done in tie-making, and in other forms of tailoring and sewing, so that the garment hangs in a particular way. Clothes made on the bias are more giving, flexible and fluid.

Bias also has another meaning, that used by Aubin, Renaud and St-Onge, the authors of ‘Detecting rater bias using person-fit statistics: A Monte Carlo simulation study’ [1]. Bias, in the context of health professions education (HPE) assessment, often refers to ‘an inclination or prejudice for or against one person or group’ in a way that is considered unfair [2]. This kind of bias is a very serious concern, not only in HPE assessment, but in many aspects of life, encompassing issues of race, gender, religion, class, ability, age and other factors. Bias can also refer to a focus on a topic or a distortion of statistics, which is also relevant for HPE research.

Aubin, Renaud and St-Onge are particularly interested in rater bias, which they point out is becoming increasingly relevant in the context of performance-based assessment which relies on observing and scoring performances. In such assessments, the subjectivity of raters is brought to the fore. Aubin, Renaud and St-Onge do not say so explicitly, but seem to align themselves with those who view this subjectivity as ‘a source of measurement error’. The subjectivity of examiners is described as being influenced by the examinee’s prior performances, values, work habits and demographics, which taint the attributed scores in that the measurements ‘do not strictly reflect their performance’. Rater bias is considered to reduce the validity of score interpretation in assessment.

There has been a push recently in HPE to reconsider what it means to measure examinees’ performances using scores, with arguments for an increased recognition of the power and value of qualitative assessments [3, 4]. In these discussions, subjectivity is embraced rather than regarded as tainting outcomes [5]. These discussions about subjectivity are important, yet, as I am currently exploring with colleagues, a stronger embrace with the subjective is not clear cut. Objectivity and subjectivity are very hard categories to keep separate. In fact, it is the attempt to keep them separate that is so interesting.

In the social science disciplines in which I work as a researcher studying medical education—anthropology and science and technology studies—facts are not considered to be inherent truths but achievements brought about by practices of various actors (human and non-human). A nice example is the disease atherosclerosis. The ethnographer and philosopher Annemarie Mol [6] shows that atherosclerosis is not just one thing, but many things. That is, it has multiple forms, whether you look at the practices of physicians, who might work with atherosclerosis as a series of symptoms and signs, or pathologists, who see a histological formation under a microscope, or the patient, who may experience atherosclerosis as pain in the leg. Atherosclerosis is also manifest through other tests and measurements. It is thus brought about (Mol uses the word ‘enacted’) through the practices of history telling, slide preparations, ankle-brachial index scores and angiographies. Atherosclerosis does not exist of and by itself, it is enacted through these practices.

Bias can be considered similarly. Bias is not an inherent fact, but rather something that is constructed. What it means to be subjective or objective is construed by different communities of practice. Valid scores, unbiased ratings, coherence and agreement between examiners is an outcome, not something that exists in a pure form. As a social scientist I am not interested in whether bias exists or not, or how to remove it, but rather in the practices of those who are attempting to detect and measure, or work with it, in some way.

This is why I found Aubin, Renaud and St-Onge’s article so fascinating. The authors propose an extremely elegant model for calculating rater bias, one that attends to specificity and sensitivity of statistics due to it not being conducted in a ‘naturalistic setting’. This Monte Carlo simulation methodology using *l*_*z*_ statistics generates data that represent rater-based assessment in a controlled setting, designed to mimic types of bias. The aim of designing such a simulation is to help identify raters who may need help or remediation, that is, those examiners whose ratings must be interpreted with caution.

If we return to the sartorial meaning of the word bias, that is, working with fabric on the oblique, we come to the essence of the problem with bias in assessment ratings, which is that it provides scoring which is too fluid, too giving, too flexible to the conditions. It means that there are too many ‘creative’ and ‘aberrant’ raters who overly attend to the individual specifics of the examinee and the situation.

However, we must always remember that bias, and the subjectivities considered to make its form, is an achievement, not a fact. Aubin, Renaud and St-Onge construct what they mean by bias in the design of their simulation. They write: ‘raters that yield* l*_*z*_ scores greater than the cutoff values are identified as biased’. Such definitions, the simulation software, the hardware, the researchers’ ‘prior performances, values, work habits and demographics’ all shape the research outcomes. Our methods matter, and always influence—or ‘enact’—what we find. This makes it important and insightful to engage seriously with the work of those who conduct research using different methodologies, and in doing so, to also look obliquely at our own research practices.
